# Obesity and Cardiac Conduction Block Disease in China

**DOI:** 10.1001/jamanetworkopen.2023.42831

**Published:** 2023-11-13

**Authors:** Peipei Liu, Yanxiu Wang, Xiaofu Zhang, Zihao Zhang, NaiHui Zhao, Wenli Ou, Guodong Wang, Xuemei Yang, Man Li, Yaya Zhang, Xiuhong Yang, Shouling Wu

**Affiliations:** 1 School of Public Health, North China University of Science and Technology, Caofeidian Eco-city, Tangshan, Hebei, China; 2 Department of Cardiology, Kailuan General Hospital, Tangshan, Hebei, China; 3 Hebei Key Laboratory for Chronic Diseases, Tangshan Key Laboratory for Preclinical and Basic Research on Chronic Diseases, School of Basic Medical Sciences, North China University of Science and Technology, Caofeidian Eco-city, Tangshan, Hebei, China; 4 School of Clinical Medicine, North China University of Science and Technology, Caofeidian Eco-city, Tangshan, Hebei, China

## Abstract

**Importance:**

Although a high body mass index (BMI) has been found to be associated with increased risk of cardiac conduction block (CCB) in older adults, no further studies have investigated the association between obesity and CCB in the general population.

**Objective:**

To investigate the association between obesity and CCB, including its subtypes.

**Design, Setting, and Participants:**

This cohort study used data from participants in the Kailuan Study in China (2006-2018) who had completed a physical examination in 2006 (baseline) and had not experienced CCB before baseline. Data analysis was conducted from March to September 2023.

**Exposures:**

Obesity status was defined by BMI in 3 groups: normal weight (18.5 to <24), overweight (24 to <28), and obesity (≥28).

**Main Outcome and Measures:**

The primary outcome was CCB, which was diagnosed from standard 12-lead electrocardiography. The primary end point included high-grade atrioventricular block (HAVB), complete right bundle branch block, complete left bundle branch block, left anterior fascicular block (LAFB), and left posterior fascicular block. First-degree atrioventricular block (FAVB), second-degree type 1 AVB, HAVB, complete and incomplete right and left bundle branch block, LAFB, and left posterior fascicular block were considered separately as secondary end points.

**Results:**

Among 86 635 participants (mean [SD] age, 50.8 [11.9] years; 68 205 males [78.7%]), there were 33 259 individuals with normal weight (38.4%), 37 069 individuals with overweight (42.8%), and 16 307 individuals with obesity (18.8%). The mean (SD) follow-up was 10.6 (3.07) years. In the multivariable Cox proportional hazards regression analysis, obesity was associated with an increased risk of incident CCB (hazard ratio [HR], 1.21; 95% CI, 1.04-1.42) vs normal BMI. In secondary analysis, obesity was associated with an increased risk of FAVB (HR, 1.44; 95% CI, 1.21-1.73), HAVB (HR, 1.99; 95% CI, 1.03-3.82), and LAFB (HR, 1.29; 95% CI, 1.03-1.62) vs normal BMI. There was no association between obesity and other CCB subtypes. Obesity was associated with a greater increase in risk of CCB vs normal BMI in older (aged ≥65 years; HR, 1.44; 95% CI, 1.05-1.96) vs younger (aged <65 years; HR, 1.13; 95% CI, 0.96-1.34) participants (*P* for interaction < .001) and those with diabetes (HR, 2.16; 95% CI, 1.24-3.76) vs without diabetes (HR, 1.19; 95% CI, 1.02-1.39) (P for interaction = .02).

**Conclusions and Relevance:**

This study found that obesity was associated with an increased risk of CCB, with greater increases in risk for FAVB, HAVB, and LAFB. Individuals who were older and those who had diabetes had larger increases in risk.

## Key Points

**Question:**

Is obesity associated with cardiac conduction disease?

**Findings:**

In this cohort study of 86 635 individuals without cardiac conduction block at baseline, those with obesity were at a higher risk of developing cardiac conduction diseases, particularly atrioventricular block. This increase in risk was greater among individuals who were older and those with diabetes.

**Meaning:**

These findings suggest that control of body mass may be associated with reduced risk of cardiac conduction disease.

## Teaser

This cohort study investigates the association of obesity with risk of developing cardiac conduction block and subtypes among patients in China.

## Introduction

Cardiovascular diseases are the leading causes of global morbidity and mortality.^1,2^ In 2020, approximately 19 million people were estimated to have died from cardiovascular diseases.^3^ Arrhythmia is a common type of cardiovascular disease.^4^ According to the most recent statistics released by the American College of Cardiology (ACC), American Heart Association (AHA), and Heart Rhythm Society (HRS), arrhythmia-induced sudden cardiac death accounts for 20% to 50% of all cardiovascular-related fatalities.^5^ Although the pathogenesis of bradycardia and tachycardia differs, both lead to adverse clinical outcomes.^6,7,8,9^ Cardiac conduction block (CCB) is characterized by slow, excessively slow, or even complete blocked pulse conduction, which can have severe consequences.^10^ Even a seemingly simple first-degree atrioventricular block (FAVB) or bundle branch block is associated with increased risk of cardiovascular adverse events, such as heart failure^7^ and all-cause mortality.^8^ Therefore, the identification of modifiable factors associated with high risk of CCB is crucial. Established risk factors associated with CCB include hypertension,^11^ diabetes,^12^ and inflammation.^13^

Obesity is a significant risk factor associated with adverse cardiovascular events.^14^ Every 5-point increase in body mass index (BMI; calculated as weight in kilograms divided by height in meters squared)^15^ could contribute to a 16% increase in risk of sudden cardiac death^16^ and 29% increase in risk of atrial fibrillation.^17^ Most previous studies have primarily examined the association between obesity and tachyarrhythmias, such as atrial fibrillation^18^ and ventricular arrhythmia.^19^ However, few studies have investigated the association between obesity and bradyarrhythmias. In a 2023 study investigating the association between lifestyle habits and CCB, Frimodt-Møller et al^20^ found that a high BMI was associated with an increased risk of CCB in older adults. However, no study, to our knowledge, has reported the association between obesity and CCB in the general population. Therefore, we used data from the Kailuan Study cohort^21^ to investigate the association between obesity and CCB and its subtypes, such as high-grade AVB (HAVB).

## Methods

This cohort study was conducted in accordance with the principles of the Declaration of Helsinki and approved by the Ethics Committee of Kailuan General Hospital. All participants or their legal representatives provided written informed consent. This study is reported following the Strengthening the Reporting of Observational Studies in Epidemiology (STROBE) reporting guideline.

### Study Sample

The study sample was drawn from the Kailuan Study, an ongoing prospective cohort study initiated in 2006. Active and retired employees of the Kailuan Group underwent comprehensive assessments in 2006 at Kailuan General Hospital in China and its 11 affiliated hospitals. These assessments included anthropometric measurements (such as height and weight), electrocardiographic evaluations, questionnaire surveys, and laboratory testing. Subsequent follow-up visits occurred biennially.

**Figure 1.**
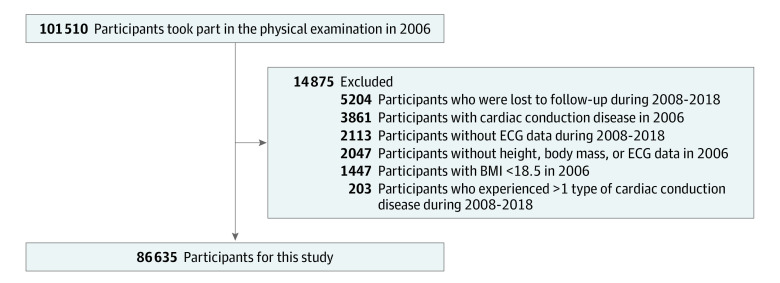
Study Flowchart BMI indicates body mass index (calculated as weight in kilograms divided by height in meters squared); ECG, electrocardiogram.

We established the baseline using data collected during 2006 assessments. Participants were excluded if they lacked complete data on height, body mass, or electrocardiograms (ECGs); had a BMI less than 18.5; had preexisting CCB at the baseline assessment; had missing ECG data during the follow-up or were lost to follow-up; or experienced more than 1 type of CCB disease (Figure 1).

### Data Collection

The epidemiological investigation and anthropometric and biochemical methods used have been described in detail previously .^22^ Height, body mass, and other pertinent measurements were collected by medically trained personnel in strict accordance with standard protocols. According to the protocol, participants stood barefoot in lightweight clothing with arms relaxed while standard instruments were used to measure standing height and body mass to precisions of 0.1 cm and 0.1 kg, respectively.^15^ According to the criteria of the Chinese Working Group on Obesity, BMI is categorized into the following 3 groups: normal weight (18.5-24), overweight (24 to <28), and obesity (≥28).^23^

During the 2006 physical examination and subsequent biennial follow-up, participants underwent standard 12-lead ECG examination. Between 7 am and 9 am on the examination day after a period of complete rest, participants underwent 10-second 12-lead ECG examination. ECG measurements and diagnoses were made by at least 2 trained electrocardiologists. Blockade events were diagnosed in accordance with ACC/AHA/HRS practice guidelines. CCB is defined as the occurrence of any of the following: HAVB, complete right bundle branch block (RBBB), complete left bundle branch block (LBBB), left anterior fascicular block (LAFB), or left posterior fascicular block (LPFB). HAVB is defined as second-degree type 2 AVB, third-degree AVB, or pacemaker status due to AVB. The pacemaker status and reasons for pacemaker placement were obtained through an electronic medical information system query. Diagnostic criteria are listed in more detail in eTable 1 in Supplement 1.

Low-density lipoprotein cholesterol (LDL-C), high-density lipoprotein cholesterol (HDL-C), creatinine, and high-sensitivity C-reactive protein (hs-CRP) were measured by auto analyzer (Hitachi 747; Hitachi). Estimated glomerular filtration rate (eGFR) was calculated from creatinine following the Chronic Kidney Disease Epidemiology Collaboration formula.^24^

### Follow-Up and End Point Events

The 2006 physical examination marked the beginning of the follow-up period. The primary outcome was CCB, which included HAVB, complete RBBB, complete LBBB, LAFB, LPFB, and death, whichever happened first. The secondary analysis separately considered end points of FAVB, second-degree type 1 AVB, HAVB, complete RBBB, incomplete RBBB, complete LBBB, incomplete LBBB, LAFB, LPFB, and death. The follow-up period ended on December 31, 2019.

### Definitions

Factors included hypertension, diabetes, drinking, smoking, physical exercise, high salt intake, myocardial infarction during follow-up, and heart failure during follow-up. Definitions can be found in the eMethods in Supplement 1.

### Statistical Analysis

Normally distributed continuous variables are presented as the mean (SD) and compared using 1-way analysis of variance with the Tukey post hoc test. Nonnormally distributed continuous variables are presented as the median with IQR and compared using the Kruskal-Wallis test. Categorical variables are presented as percentages and groups compared using the χ^2^ test.

Cox proportional hazards regression models were used to assess the association of BMI group with CCB and its subtypes by calculating the hazard ratio (HR) and corresponding 95% CI. In the primary analysis, the end point was defined as CCB, which included HAVB, complete RBBB, complete LBBB, LAFB, and LPFB. In the secondary analysis, we further examined each of the following end points separately: FAVB, HAVB, complete RBBB, incomplete RBBB, complete LBBB, incomplete LBBB, and LAFB. The normal-weight group served as the reference group, and the multivariable model was sequentially adjusted for age, sex, smoking (yes or no), drinking (yes or no), physical exercise (yes or no), high salt intake (>6 g/d; yes or no), LDL-C level, HDL-C level, eGFR, hs-CRP level (log transformed), hypertension (yes or no), diabetes (yes or no), incident heart failure and myocardial infarction during the follow-up period (yes or no), and the use of antidiabetic treatment, antihypertensive treatment, or lipid-lowering drugs (yes or no). A restricted cubic spline function was used to assess the dose-response association between BMI and the risk of CCB, with 3 nodes at the twenty-fifth, fiftieth, and seventy-fifth percentiles.

Multiplicative terms were added to Cox proportional hazards regression models to assess potential interaction associations between BMI group and potential effect modifiers. Stratified analyses were conducted accordingly. To assess the robustness of our results, we conducted sensitivity analyses as follows: first, we divided the entire follow-up period into six 2-year intervals. A time-dependent Cox model was used to assess the association between BMI, which was updated over time, and CCB adjusted for time-dependent covariates. Second, we excluded individuals who developed CCB within 2 years of follow-up. Third, we sequentially excluded individuals who experienced heart failure or myocardial infarction events during the follow-up period. Last, we performed competing risk regression (Fine-Gray model) to address potential mortality-related confounding. Data analysis was conducted from March 2023 to September 2023. Statistical analyses were performed using SAS statistical software version 9.4 (SAS Institute), and statistical significance was accepted at a 2-tailed *P* < .05.

## Results

Initially, a total of 101 510 participants underwent a baseline physical examination in 2006. After excluding 2047 participants due to missing height, body mass, or ECG data; 1447 participants with BMI less than the normal (18.5) cutoff; 3861 participants who had a CCB at baseline; 2113 participants for whom ECG data during follow-up were unavailable; 5204 participants who were lost to follow-up; and 203 participants who had experienced more than 1 CCB disease, a total of 86 635 participants (mean [SD] age, 50.8 [11.9] years; 68 205 males [78.7%]) remained in the analysis cohort (Figure 1).

The mean (SD) BMI was 25.2 (3.4). Baseline characteristics of participants by BMI group are summarized in Table 1. There were 33 259 individuals with normal weight (38.4%), 37 069 individuals with overweight (42.8%), and 16 307 individuals with obesity (18.8%). Compared with individuals with normal weight, those in the obesity group had higher baseline levels of LDL-C and hs-CRP. They were also more likely to take antihypertensive, hypoglycemic, and lipid-lowering drugs and had a higher prevalence of hypertension and diabetes. Additionally, a larger proportion of individuals with obesity engaged in regular physical activity and had high salt intake, and they had lower HDL-C and eGFR levels. Furthermore, during the follow-up period, the obesity group experienced higher rates of heart failure (560 individuals [3.43%] vs 601 individuals [1.81%]; *P* < .001) and myocardial infarction (339 individuals [2.08%] vs 383 individuals [1.15%]; P < .001) compared with the normal weight group (Table 1).

**Table 1.** Participant Baseline Characteristics

Characteristic	Participants, No. (%)				
	Total (N = 86 635)	BMI 18.5 to <24 (n = 33 259)	BMI 24 to <28 (n = 37 069)	BMI ≥28 (n = 16 307)	*P* value^a^
Age, mean (SD), year	50.8 (11.9)	50.0 (12.7)	51.4 (11.3)	50.7 (11.7)	<.001
Sex					
Female	18 430 (21.3)	8408 (25.3)	6773 (18.3)	3249 (19.9)	<.001
Male	68 205 (78.7)	24 851 (74.7)	30 296 (81.7)	13 058 (80.1)	
Current smoker	29 846 (34.5)	11 582 (34.8)	12 897 (34.8)	5367 (32.9)	<.001
Current drinker	2521 (2.91)	964 (2.90)	1080 (2.91)	477 (2.93)	.90
Physical activity	13 482 (15.6)	4970 (14.9)	5891 (15.9)	2621 (16.1)	<.001
High salt intake	9393 (10.8)	3212 (9.66)	4139 (11.2)	2042 (12.5)	<.001
Hs-CRP, median (IQR), mg/dL	0.08 (0.03-0.22)	0.06 (0.02-0.17)	0.09 (0.03-0.22)	0.12 (0.05-0.30)	<.001
HDL-C, mean (SD) mg/dL	59.46 (15.06)	61.39 (15.44)	58.69 (14.67)	57.53 (14.67)	<.001
LDL-C, mean (SD), mg/dL	90.73 (33.98)	88.03 (333.59)	92.28 (33.98)	93.44 (34.36)	<.001
eGFR, mean (SD), ml/min/1.73 m^2^	82.6 (22.4)	84.8 (22.2)	81.6 (22.5)	81.5 (22.4)	<.001
BMI, mean (SD)	25.2 (3.35)	21.9 (1.41)	25.8 (1.13)	30.2 (2.23)	<.001
Hypertension	35 888 (41.4)	9843 (29.6)	16 660 (44.9)	9385 (57.6)	<.001
Diabetes	7789 (8.99)	1893 (5.69)	3746 (10.1)	2150 (13.2)	<.001
During follow-up					
Myocardial infarction	1311 (1.51)	383 (1.15)	589 (1.59)	339 (2.08)	<.001
Heart failure	2035 (2.35)	601 (1.81)	874 (2.36)	560 (3.43)	<.001
Antihypertensive treatment	9218 (10.6)	1943 (5.84)	4340 (11.7)	2935 (18.0)	<.001
Antidiabetic treatment	1950 (2.25)	479 (1.44)	941 (2.54)	530 (3.25)	<.001
Lipid-lowering drug	791 (0.91)	175 (0.53)	368 (0.99)	248 (1.52)	<.001

Abbreviations: BMI, body mass index (calculated as weight in kilograms divided by height in meters squared); eGFR, estimated glomerular filtration rate; HDL-C, high-density lipoprotein cholesterol; hs-CRP, high-sensitivity C-reactive protein; LDL-C, low-density lipoprotein cholesterol.

SI conversion factors: To convert HDL-C and LDL-C to millimoles per liter, multiply by 0.0259; hs-CRP to milligrams per liter, multiply by 10.

^a^ Comparison of baseline characteristics between BMI groups.

During a mean (SD) follow-up of 10.6 (3.07) years, there were 1258 new cases of CCB among all participants, corresponding to an incidence of 1.57 incidents per 10 000 person-years. For subtypes, there were 943 incidents of FAVB, 9 incidents of second-degree type 1 AVB, 71 incidents of HAVB, 568 incidents of complete RBBB, 850 incidents of incomplete RBBB, 40 incidents of complete LBBB, 27 incidents of incomplete LBBB, 570 incidents of LAFB, and 14 incidents of LPFB. Cumulative incidence rates of CCB across BMI groups are presented in eFigure 1 in Supplement 1. Additionally, cubic spline models indicated a linear association between BMI and the risk of CCB (eFigure 2 in Supplement 1).

In Cox proportional hazards regression multivariable analysis, obesity was associated with an increased risk of CCB. Compared with the normal weight group (reference), the multivariable adjusted HR was 1.03 (95% CI, 0.91-1.17) for the overweight group and 1.21 (95% CI, 1.04-1.42) for the obesity group (Table 2). For every 5-point increase in BMI, the multivariable adjusted HR was 1.11 (95% CI, 1.02-1.21) for CCB (Table 2). In the secondary analysis, obesity was associated with an increased risk of FAVB (adjusted HR, 1.44; 95% CI, 1.21-1.73), HAVB (adjusted HR, 1.99; 95% CI, 1.03-3.82), and LAFB (HR, 1.29; 95% CI, 1.03-1.62) compared with normal BMI (Table 3). Higher BMI was also associated with an increased risk of incomplete RBBB (adjusted HR per 5-unit increase, 1.13; 95% CI, 1.02-1.25) and LAFB (adjusted HR per 5-unit increase, 1.19; 95% CI, 1.05-1.33) (eTable 2 in Supplement 1). Among other CCB subtypes, there was no association between obesity and complete RBBB, complete LBBB, or incomplete LBBB (eTable 2 in Supplement 1).

**Table 2.** Association of BMI and CCB

Model^a^	CCB, HR (95% CI) (N = 86 635)^b^			
	BMI 18.5 to <24 (n = 33 259)	BMI 24 to <28 (n = 37 069)	BMI ≥28 (n = 16 307)	Per 5-unit increase
CCB, No (%)	438 (1.32)	546 (1.47)	274 (1.68)	NA
Incidence rate, No./10 000 person-y	12.38	13.90	12.89	NA
Model 1	1 [Reference]	1.05 (0.93-1.19)	1.26 (1.09-1.47)	1.14 (1.05-1.23)
Model 2	1 [Reference]	1.04 (0.91-1.18)	1.23 (1.05-1.43)	1.11 (1.02-1.21)
Model 3	1 [Reference]	1.03 (0.91-1.17)	1.22 (1.04-1.42)	1.11 (1.02-1.21)
Model 4	1 [Reference]	1.03 (0.91-1.17)	1.21 (1.04-1.42)	1.11 (1.02-1.21)

Abbreviations: BMI, body mass index (calculated as weight in kilograms divided by height in meters squared); CCB, cardiac conduction block; HR, hazard ratio; NA, not applicable.

^a^ Model 1 adjusted for age and sex. Model 2 adjusted for age, sex, smoking, drinking, high salt intake, physical activity, high-sensitivity C-reactive protein level, low-density lipoprotein cholesterol level, high-density lipoprotein cholesterol level, estimated glomerular filtration rate, hypertension, and diabetes. Model 3 adjusted for all variables in model 2 and myocardial infarction during follow-up and heart failure during follow-up. Model 4 adjusted for all variables in model 3 and antidiabetic treatment, antihypertensive treatment, and lipid-lowering drug use.

^b^ CCB is defined as the occurrence of any of the following: high-grade atrioventricular block, complete right bundle branch block, complete left bundle branch block, left anterior fascicular block, or left posterior fascicular block.

**Table 3.** Association of BMI and AVB

Model^a^	AVB, HR (95% CI) (N = 86 635)			
	BMI 18.5 to <24 (n = 33 259)	BMI 24 to <28 (n = 37 069)	BMI ≥28 (n = 16 307)	Per 5-unit increase
**FAVB**				
No. (%)	309 (0.93)	410 (1.11)	224 (1.37)	NA
Incidence rate, No./10 000 person-y	8.72	10.42	12.98	NA
Model 1	1 [Reference]	1.13 (0.97-1.31)	1.47 (1.24-1.75)	1.26 (1.14-1.38)
Model 2	1 [Reference]	1.15 (0.99-1.34)	1.51 (1.26-1.80)	1.28 (1.16-1.41)
Model 3	1 [Reference]	1.15 (0.99-1.33)	1.51 (1.27-1.81)	1.28 (1.16-1.41)
Model 4	1 [Reference]	1.12 (0.97-1.30)	1.44 (1.21-1.73)	1.26 (1.14-1.39)
**HAVB** ^b^				
No. (%)	20 (0.06)	32 (0.09)	19 (0.12)	NA
Incidence rate, No./10 000 person-y	0.56	0.81	1.09	NA
Model 1	1 [Reference]	1.42 (0.80-2.51)	1.99 (1.05-3.77)	1.33 (0.95-1.87)
Model 2	1 [Reference]	1.43 (0.81-2.54)	2.04 (1.06-3.91)	1.34 (0.95-1.90)
Model 3	1 [Reference]	1.43 (0.80-2.53)	2.03 (1.05-3.90)	1.34 (0.94-1.89)
Model 4	1 [Reference]	1.42 (0.80-2.52)	1.99 (1.03-3.82)	1.32 (0.93-1.87)

Abbreviations: AVB, atrioventricular block; BMI, body mass index (calculated as weight in kilograms divided by height in meters squared); FAVB, first-degree atrioventricular block; HAVB, high-grade atrioventricular block; HR, hazard ratio; NA, not applicable.

^a^ Model 1 adjusted for age and sex. Model 2 adjusted for age, sex, smoking, drinking, high salt intake, physical activity, high-sensitivity C-reactive protein level, low-density lipoprotein cholesterol level, high-density lipoprotein cholesterol level, estimated glomerular filtration rate, hypertension, and diabetes. Model 3 adjusted for all variables in model 2 and myocardial infarction during follow-up and heart failure during follow-up. Model 4 adjusted for all variables in model 3 and antidiabetic treatment, antihypertensive treatment, and lipid-lowering drug use.

^b^ HAVB is defined as second-degree type 2 AVB, third-degree AVB, or pacemaker use due to AVB.

**Figure 2.**
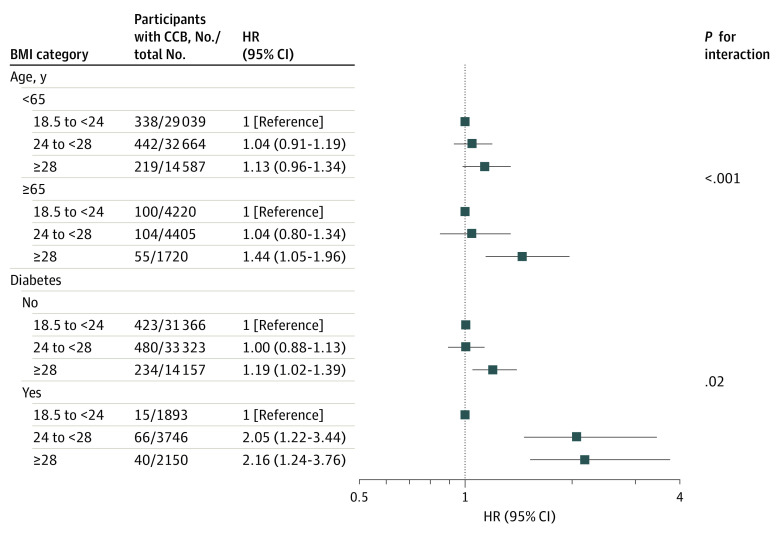
Risk of Cardiac Conduction Block (CCB) by Body Mass Index (BMI) Group BMI is calculated as weight in kilograms divided by height in meters squared. Boxes indicate hazard ratios (HRs); whiskers, 95% CIs.

We observed interactions between age and diabetes and the association of BMI with risk of CCB. Obesity was associated with a greater increase in risk of CCB compared with normal BMI in participants with (HR, 2.16; 95% CI, 1.24-3.76) vs without (HR, 1.19; 95% CI, 1.02-1.39) diabetes (97 percentage points higher; *P* for interaction = .02) and older (aged ≥65 years; HR, 1.44; 95% CI, 1.05-1.96) vs younger (aged <65 years; HR, 1.13; 95% CI, 0.96-1.34) individuals (*P* for interaction < .001) (Figure 2).

To validate the stability of our results, we conducted a series of sensitivity analyses using time-varying BMI as the exposure (eTable 3 in Supplement 1), excluding individuals who developed CCB within 2 years of follow-up (eTable 4 in Supplement 1), and sequentially excluding individuals who experienced heart failure or myocardial infarction events during the follow-up period (eTable 4 in Supplement 1). Results did not materially change. Additionally, the Fine-Grey model for competing mortality risk yielded results consistent with primary results (eTable 5 in Supplement 1).

## Discussion

The principal finding of this cohort study was that obesity was associated with an increased risk of CCB, especially that of AVB. In addition, the associated increase in risk was notably larger in older individuals and those with diabetes.

We found that obesity was a risk factor associated with CCB, and there was a linear, positive association between BMI and the onset of CCB. To our knowledge, this is the first large prospective cohort study on obesity and CCB in a community-dwelling, general population. Previous research by Frimodt-Moller et al^20^ found an HR of 1.22 (95% CI, 1.06-1.41) for CCB with each 10-point increase in BMI in a study involving 4381 individuals aged older than 65 years. A 1986 cross-sectional study^25^ of 1029 individuals with obesity yielded similar results. Frank et al^25^ identified positive associations between increased obesity and prolonged PR interval, widened QRS complex, and lengthened QTc interval. The Atherosclerosis Risk in Communities Study^26^ also reported a positive association between BMI and the risk of PR interval prolongation. Compared with previous studies, our research includes a wider range of participant ages, and unlike previous studies, we performed multiple analyses for robustness, including time-dependent Cox analysis, competing risk analysis for mortality risk, and sensitivity analyses. Our research extends the evidence base by finding that obesity was an independent risk factor associated with the incidence of CCB, thus contributing novel insights to the association between obesity and cardiovascular diseases.

Notably, we observed specific associations between obesity and a few CCB subtypes. Individuals with obesity (BMI >28) had a markedly higher risk of developing HAVB and FAVB, with risks increased by 99% and 44%, respectively, compared with the normal weight group. Meanwhile, for every 5-point increase in BMI, there was a 19% increased risk of developing LAFB and a 13% increased risk of developing incomplete RBBB. For other subtypes, the HR comparing obesity vs normal weight group were all nonsignificant. Although we observed these differences, our study is observational and thus unable to analyze underlying mechanisms. Nonsignifcant outcomes for these groups may be due to smaller case numbers of subtypes such as LPFB, complete LBBB, and incomplete LBBB. We also speculate potential mechanisms. First, in contrast to the dual blood supply of left and right bundle branches, the atrioventricular node receives its blood supply from only the atrioventricular node artery, which originates from the right coronary artery. Right coronary artery occlusion often causes first- to third-degree AVB,^27,28^ and obesity is positively associated with the risk and severity of coronary artery disease.^29^ Second, left and right bundle branches have a fan-shaped structure with many branches, which renders them less susceptible to occlusion, whereas the Bachmann 3 branches may be more vulnerable to metabolic disturbances.^30,31^ Third, left and right bundle branches pass through the ventricular myocardium, which may expose them to a more substantial influence of hemodynamics.^30^ Last, another study by Frimodt-Moller et al^32^ indirectly corroborated our findings by demonstrating that intensive blood pressure control was associated with reduced risk of LBBB but not RBBB. Obesity, by increasing preload, places additional strain on the heart and can result in left ventricular hypertrophy, a condition that may trigger interstitial fibrosis.^33^ This fibrosis, in turn, has the potential to downregulate gap junctions, disrupting the normal coupling of cardiac cells.^34^

Furthermore, we observed that diabetes status had an interaction with the association between obesity and CCB. Stratification analysis by diabetes status (yes or no) revealed that the risk of CCB associated with obesity in individuals with diabetes was 97 percentage points higher than in those without diabetes. This finding aligns with the result of Haxha et al^12^ that type 2 diabetes was associated with a higher rate of third-degree AVB compared with matched individuals in a control group. Several factors likely interact with these associations. For example, diabetes is associated with electrolyte imbalances, which have the potential to disrupt cardiac repolarization.^35,36^ Moreover, diabetes may be associated with cardiac autonomic neuropathy, which is associated with sympathetic and parasympathetic systems.^37^ We observed an interaction association for age. The risk of CCB associated with obesity was observed only among older individuals (ages ≥65 years). Previous research found that older age was associated with incident conduction disease.^20,38^ Age-related degenerative changes in the heart, along with the increased prevalence of conditions such as hypertension, diabetes, and coronary artery disease that come with aging, may contribute to the increased risk of cardiac conduction disorders.^39^ Additionally, there may be potential synergistic interactions between obesity and these factors. These findings suggest that special attention should be given to the cardiovascular health of individuals with obesity, particularly older individuals with diabetes.

Our findings have significant public health implications for the prevention of CCB, a disease that is associated with multiple adverse cardiovascular outcomes and all-cause mortality.^6,7,8,40,41^ Severe AVB has a poor prognosis, and although cardiac pacemakers are an effective treatment, their high cost and risk of infection place substantial economic and disease burdens on patients. This study found that obesity was associated with an increased risk of CCB, especially AVB, but it is a modifiable and reversable risk factor. Previous studies found that weight loss was associated with reductions in risks of endometrial cancer by 56%,^42^ cardiovascular disease by 11%,^43^ and all-cause mortality by 18%.^44^ These findings suggest that if patients reduce their weight through exercise and dietary measures, they may be highly likely to mitigate the risk of CCB and avoid the economic burden associated with the implantation of a cardiac pacemaker.

### Strengths and Limitations

Strengths of this study include its prospective nature, large sample size, long-term follow-up, and use of comprehensive biological data. Furthermore, body mass and height data were collected by direct measurement rather than self-reports and so may have greater accuracy. In addition, events were identified on routine ECG rather than during hospitalization, minimizing the risk of missing participants who were asymptomatic.

However, this study also had several limitations. First, owing to the study’s observational nature, causal relationships between obesity and CCB cannot be ascribed. The identified associations may reflect the overall association of obesity with cardiovascular health, rather than a direct association with CCB. However, a sensitivity analysis found that the exclusion of participants with heart failure or myocardial infarction during follow-up did not significantly alter results. Second, details of participant medical history, smoking habits, alcohol consumption habits, and physical exercise habits were obtained through self-reporting at baseline, which may have led to recall bias. However, consistent results obtained from time-dependent Cox regression analysis support the validity of our findings. Third, it is worth noting that our findings may have limited generalizability given that our study was conducted exclusively with Chinese workers and lacks validation in non-Asian populations.

## Conclusions

This cohort study found an association between obesity and increased risk of CCB. The increase in risk was higher among older individuals and those with diabetes.

## Notes

Supplement 1. **eTable 1.** Definitions of Cardiac Conduction Block Diseases **eTable 2.** Association Between Body Mass Index and Cardiac Conduction Block Subtypes **eTable 3.** Association Between Body Mass Index and Cardiac Conduction Block Using Time-Dependent Cox Proportional Hazards Model **eTable 4.** Association Between Body Mass Index and Cardiac Conduction Block, Sensitivity Analyses **eTable 5.** Association Between Body Mass Index and Cardiac Conduction Block Using Fine-Gray Model **eFigure 1.** Cumulative Incidences for Cardiac Conduction Block **eFigure 2.** Dose-Response Association Between Body Mass Index and Cardiac Conduction Block **eMethods.** Definitions

Supplement 2.
Data Sharing Statement

## References

[1] Weir HK, Anderson RN, Coleman King SM, . Heart disease and cancer deaths—trends and projections in the United States, 1969-2020. Prev Chronic Dis. 2016;13:E157. doi:10.5888/pcd13.160211 27854420 PMC5127176

[2] Kivimäki M, Steptoe A. Effects of stress on the development and progression of cardiovascular disease. Nat Rev Cardiol. 2018;15(4):215-229. doi:10.1038/nrcardio.2017.189 29213140

[3] Tsao CW, Aday AW, Almarzooq ZI, . Heart disease and stroke statistics-2022 update: a report from the American Heart Association. Circulation. 2022;145(8):e153-e639. doi:10.1161/CIR.0000000000001052 35078371

[4] Fu DG. Cardiac arrhythmias: diagnosis, symptoms, and treatments. Cell Biochem Biophys. 2015;73(2):291-296. doi:10.1007/s12013-015-0626-4 25737133

[5] Al-Khatib SM, Stevenson WG, Ackerman MJ, . 2017 AHA/ACC/HRS guideline for management of patients with ventricular arrhythmias and the prevention of sudden cardiac death: executive summary: a report of the American College of Cardiology/American Heart Association Task Force on Clinical Practice Guidelines and the Heart Rhythm Society. Circulation. 2018;138(13):e210-e271. doi:10.1161/CIR.0000000000000548 29084733

[6] Cheng S, Keyes MJ, Larson MG, . Long-term outcomes in individuals with prolonged PR interval or first-degree atrioventricular block. JAMA. 2009;301(24):2571-2577. doi:10.1001/jama.2009.888 19549974 PMC2765917

[7] Crisel RK, Farzaneh-Far R, Na B, Whooley MA. First-degree atrioventricular block is associated with heart failure and death in persons with stable coronary artery disease: data from the Heart and Soul Study. Eur Heart J. 2011;32(15):1875-1880. doi:10.1093/eurheartj/ehr139 21606074 PMC3202329

[8] Aro AL, Anttonen O, Kerola T, . Prognostic significance of prolonged PR interval in the general population. Eur Heart J. 2014;35(2):123-129. doi:10.1093/eurheartj/eht176 23677846

[9] Vinding NE, Kristensen SL, Rørth R, . Ischemic stroke severity and mortality in patients with and without atrial fibrillation. J Am Heart Assoc. 2022;11(4):e022638. doi:10.1161/JAHA.121.022638 35156393 PMC9245802

[10] Smits JP, Veldkamp MW, Wilde AA. Mechanisms of inherited cardiac conduction disease. Europace. 2005;7(2):122-137. doi:10.1016/j.eupc.2004.11.004 15763526

[11] Kerola T, Eranti A, Aro AL, . Risk factors associated with atrioventricular block. JAMA Netw Open. 2019;2(5):e194176. doi:10.1001/jamanetworkopen.2019.4176 31125096 PMC6632153

[12] Haxha S, Halili A, Malmborg M, . Type 2 diabetes mellitus and higher rate of complete atrioventricular block: a Danish nationwide registry. Eur Heart J. 2023;44(9):752-761. doi:10.1093/eurheartj/ehac662 36433808

[13] Yang X, Zhao S, Wang S, . Systemic inflammation indicators and risk of incident arrhythmias in 478,524 individuals: evidence from the UK Biobank cohort. BMC Med. 2023;21(1):76. doi:10.1186/s12916-023-02770-5 36855116 PMC9976398

[14] Ernault AC, Meijborg VMF, Coronel R. Modulation of cardiac arrhythmogenesis by epicardial adipose tissue: JACC state-of-the-art review. J Am Coll Cardiol. 2021;78(17):1730-1745. doi:10.1016/j.jacc.2021.08.037 34674819

[15] Jia Z, Zhou Y, Liu X, . Comparison of different anthropometric measures as predictors of diabetes incidence in a Chinese population. Diabetes Res Clin Pract. 2011;92(2):265-271. doi:10.1016/j.diabres.2011.01.021 21334088

[16] Aune D, Schlesinger S, Norat T, Riboli E. Body mass index, abdominal fatness, and the risk of sudden cardiac death: a systematic review and dose-response meta-analysis of prospective studies. Eur J Epidemiol. 2018;33(8):711-722. doi:10.1007/s10654-017-0353-9 29417316 PMC6061127

[17] Wong CX, Sullivan T, Sun MT, . Obesity and the risk of incident, post-operative, and post-ablation atrial fibrillation: a meta-analysis of 626,603 individuals in 51 studies. JACC Clin Electrophysiol. 2015;1(3):139-152. doi:10.1016/j.jacep.2015.04.004 29759357

[18] Lavie CJ, Pandey A, Lau DH, Alpert MA, Sanders P. Obesity and atrial fibrillation prevalence, pathogenesis, and prognosis: effects of weight loss and exercise. J Am Coll Cardiol. 2017;70(16):2022-2035. doi:10.1016/j.jacc.2017.09.002 29025560

[19] Patel KHK, Reddy RK, Sau A, Sivanandarajah P, Ardissino M, Ng FS. Obesity as a risk factor for cardiac arrhythmias. BMJ Med. 2022;1(1):e000308. doi:10.1136/bmjmed-2022-000308 36936556 PMC9951386

[20] Frimodt-Møller EK, Soliman EZ, Kizer JR, . Lifestyle habits associated with cardiac conduction disease. Eur Heart J. 2023;44(12):1058-1066. doi:10.1093/eurheartj/ehac799 36660815 PMC10226753

[21] Zhao M, Song L, Sun L, . Associations of type 2 diabetes onset age with cardiovascular disease and mortality: the Kailuan Study. Diabetes Care. 2021;44(6):1426-1432. doi:10.2337/dc20-2375 35239970 PMC8247507

[22] Han QL, Wu SL, Liu XX, . Ideal cardiovascular health score and incident end-stage renal disease in a community-based longitudinal cohort study: the Kailuan Study. BMJ Open. 2016;6(11):e012486. doi:10.1136/bmjopen-2016-012486 27899399 PMC5168547

[23] Zhou BF; Cooperative Meta-Analysis Group of the Working Group on Obesity in China. Predictive values of body mass index and waist circumference for risk factors of certain related diseases in Chinese adults—study on optimal cut-off points of body mass index and waist circumference in Chinese adults. Biomed Environ Sci. 2002;15(1):83-96. 12046553

[24] Levey AS, Stevens LA, Schmid CH, ; CKD-EPI (Chronic Kidney Disease Epidemiology Collaboration). A new equation to estimate glomerular filtration rate. Ann Intern Med. 2009;150(9):604-612. doi:10.7326/0003-4819-150-9-200905050-00006 19414839 PMC2763564

[25] Frank S, Colliver JA, Frank A. The electrocardiogram in obesity: statistical analysis of 1,029 patients. J Am Coll Cardiol. 1986;7(2):295-299. doi:10.1016/S0735-1097(86)80494-6 3944347

[26] Magnani JW, Lopez FL, Soliman EZ, Maclehose RF, Crow RS, Alonso A. P wave indices, obesity, and the metabolic syndrome: the Atherosclerosis Risk In Communities Study. Obesity (Silver Spring). 2012;20(3):666-672. doi:10.1038/oby.2011.53 21475136 PMC3696958

[27] Anderson KR, Murphy JG. The atrio-ventricular node artery in the human heart. Angiology. 1983;34(11):711-716. doi:10.1177/000331978303401104 6638606

[28] Futami C, Tanuma K, Tanuma Y, Saito T. The arterial blood supply of the conducting system in normal human hearts. Surg Radiol Anat. 2003;25(1):42-49. doi:10.1007/s00276-002-0085-7 12819949

[29] Park JS, Ahn SG, Hwang JW, . Impact of body mass index on the relationship of epicardial adipose tissue to metabolic syndrome and coronary artery disease in an Asian population. Cardiovasc Diabetol. 2010;9:29. doi:10.1186/1475-2840-9-29 20604967 PMC2913996

[30] Sánchez-Quintana D, Yen Ho S. Anatomy of cardiac nodes and atrioventricular specialized conduction system. Anatomía de los nodos cardíacos y del sistema de conducción específico auriculoventricular. Rev Esp Cardiol. 2003;56(11):1085-1092. doi:10.1016/s0300-8932(03)77019-5 14622540

[31] Padala SK, Cabrera JA, Ellenbogen KA. Anatomy of the cardiac conduction system. Pacing Clin Electrophysiol. 2021;44(1):15-25. doi:10.1111/pace.14107 33118629

[32] Frimodt-Møller EK, Vittinghoff E, Kaur G, Biering-Sørensen T, Soliman EZ, Marcus GM. Association between intensive vs standard blood pressure control and incident left ventricular conduction disease: a post hoc analysis of the SPRINT randomized clinical trial. JAMA Cardiol. 2023;8(6):612-616. doi:10.1001/jamacardio.2023.0845 37133829 PMC10157506

[33] Alexander JK, Dennis EW, Smith WG, Amad KH, Duncan WC, Austin RC. Blood volume, cardiac output, and distribution of systemic blood flow in extreme obesity. Cardiovasc Res Cent Bull. 1962-1963;1:39-44. 14011956

[34] Spadaccio C, Rainer A, Mozetic P, . The role of extracellular matrix in age-related conduction disorders: a forgotten player? J Geriatr Cardiol. 2015;12(1):76-82. 25678907 10.11909/j.issn.1671-5411.2015.01.009 PMC4308461

[35] Yang XH, Su JB, Zhang XL, . The relationship between insulin sensitivity and heart rate-corrected QT interval in patients with type 2 diabetes. Diabetol Metab Syndr. 2017;9:69. doi:10.1186/s13098-017-0268-3 28912840 PMC5594484

[36] van Noord C, Sturkenboom MC, Straus SM, . Serum glucose and insulin are associated with QTc and RR intervals in nondiabetic elderly. Eur J Endocrinol. 2010;162(2):241-248. doi:10.1530/EJE-09-0878 19897609

[37] Vinik AI, Ziegler D. Diabetic cardiovascular autonomic neuropathy. Circulation. 2007;115(3):387-397. doi:10.1161/CIRCULATIONAHA.106.634949 17242296

[38] Eriksson P, Hansson PO, Eriksson H, Dellborg M. Bundle-branch block in a general male population: the study of men born 1913. Circulation. 1998;98(22):2494-2500. doi:10.1161/01.CIR.98.22.2494 9832497

[39] Davies M, Harris A. Pathological basis of primary heart block. Br Heart J. 1969;31(2):219-226. doi:10.1136/hrt.31.2.219 5775292 PMC487483

[40] Pantazopoulos JS, David A, Kostis WJ, Cosgrove NM, Kostis JB; Myocardial Infarction Data Acquisition System (MIDAS 30) study group. Cardiovascular outcomes in patients with intraventricular conduction blocks: a sixteen-year follow-up in a state-wide database. Hellenic J Cardiol. 2017;58(3):194-201. doi:10.1016/j.hjc.2016.11.034 27965025

[41] Tolppanen H, Siirila-Waris K, Harjola VP, . Ventricular conduction abnormalities as predictors of long-term survival in acute de novo and decompensated chronic heart failure. ESC Heart Fail. 2016;3(1):35-43. doi:10.1002/ehf2.12068 27774265 PMC5061091

[42] Luo J, Chlebowski RT, Hendryx M, . Intentional weight loss and endometrial cancer risk. J Clin Oncol. 2017;35(11):1189-1193. doi:10.1200/JCO.2016.70.5822 28165909 PMC5455602

[43] Honda T, Ishida Y, Oda M, . Changes in body weight and concurrent changes in cardiovascular risk profiles in community residents in Japan: the Hisayama study. J Atheroscler Thromb. 2022;29(2):252-267. doi:10.5551/jat.59394 33455974 PMC8803559

[44] Cook NR, Appel LJ, Whelton PK. Weight change and mortality: long-term results from the trials of hypertension prevention. J Clin Hypertens (Greenwich). 2018;20(12):1666-1673. doi:10.1111/jch.13418 30390361 PMC6289729

