# Supplementary material for: Obesity and Cardiac Conduction Block Disease in China
Source: JAMA Netw Open. 2023 Nov 13;6(11):e2342831. doi: 10.1001/jamanetworkopen.2023.42831 (PMC10644217; doi:10.1001/jamanetworkopen.2023.42831)
Supplement: Supplement 1. — eTable 1. Definitions of Cardiac Conduction Block Diseases eTable 2. Association Between Body Mass Index and Cardiac Conduction Block Subtypes eTable 3. Association Between Body Mass Index and Cardiac Conduction Block Using Time-Dependent Cox Proportional Hazards Model eTable 4. Association Between Body Mass Index and Cardiac Conduction Block, Sensitivity Analyses eTable 5. Association Between Body Mass Index and Cardiac Conduction Block Using Fine-Gray Model eFigure 1. Cumulative Incidences for Cardiac Conduction Block eFigure 2. Dose-Response Association Between Body Mass Index and Cardiac Conduction Block eMethods. Definitions [file jamanetwopen-e2342831-s001.pdf]

Supplemental Online Content

Liu P, Wang Y, Zhang X, et al. Obesity and cardiac conduction block disease in China. *JAMA Netw Open*. 2023;6(11):e2342831. doi:10.1001/jamanetworkopen.2023.42831

eTable 1. Definitions of Cardiac Conduction Block Diseases

eTable 2. Association Between Body Mass Index and Cardiac Conduction Block Subtypes

eTable 3. Association Between Body Mass Index and Cardiac Conduction Block Using Time-Dependent Cox Proportional Hazards Model

eTable 4. Association Between Body Mass Index and Cardiac Conduction Block, Sensitivity Analyses

eTable 5. Association Between Body Mass Index and Cardiac Conduction Block Using Fine-Gray Model

eFigure 1. Cumulative Incidences for Cardiac Conduction Block

eFigure 2. Dose-Response Association Between Body Mass Index and Cardiac Conduction Block

eMethods. Definitions

This supplemental material has been provided by the authors to give readers additional information about their work.

© 2023 Liu P et al. *JAMA Network Open.*

eTable 1. Definitions of the different cardiac conduction block diseases

Atrioventricular block	
First-degree atrioventricular block	P waves associated with 1:1 atrioventricular conduction and a PR interval >200 ms (this is more accurately defined as atrioventricular delay because no P waves are blocked)
Second-degree atrioventricular block	P waves with a constant rate (<100 bpm) where atrioventricular conduction is present but not 1:1
	Mobitz type I: P waves with a constant rate (<100 bpm) with a periodic single nonconducted P wave associated with P waves before and after the nonconducted P wave with inconstant PR intervals
	Mobitz type II is characterized by fixed PR intervals before and after blocked beats and is usually<br>associated with a wide QRS complex
Third-degree atrioventricular block	No evidence of atrioventricular conduction
Right BBB (RBBB)	
Complete RBBB	1. QRS duration of 120 ms or longer in the presence of normal sinus rhythm or supraventricular rhythm;<br>2.R or rSR′complex in lead V1;<br>3.rS in leads V5, V6, I, or aVL with prolonged shallow S wave.
Incomplete RBBB	Same QRS morphology criteria as complete RBBB but with a QRS duration between 110 and 119 ms
Complete LBBB	1. QRS duration of 120 ms or longer in the presence of normal sinus rhythm or supraventricular rhythm (not atrial fibrillation);<br>2. QS or rS complex in lead V1;<br>3.broad R waves in leads I, aVL, V5-V6 (or an rS pattern in V5-V6);<br>4.absence of Q waves in leads V5, V6, or I.
Incomplete LBBB	1. QRS duration between 110 and 119 ms in adults;<br>2. Presence of left ventricular hypertrophy pattern;<br>3. R peak time >60 ms in leads V4, V5, and V6;<br>4. Absence of Q wave in leads I, V5, and V6
Left anterior fascicular block	1.QRS duration <120 ms;<br>2.Frontal plane axis between −45° and −90°;<br>3.R-peak time in lead aVL of ≥45 ms;<br>4.qR (small r, tall R) pattern in lead aVL;<br>5.rS pattern (small r, deep S) in leads II, III, and aVF
Left posterior fascicular block	1. QRS duration <120 ms;<br>2. Frontal plane axis between 90° and 180° in adults;<br>3.rS (small r, deep S) pattern in leads I and aVL;<br>4. qR (small q, tall R) pattern in leads III and aVF

© 2023 Liu P et al. *JAMA Network Open.*

eTable 2. Hazard ratio and 95% confidence interval for the association between different body mass index (BMI) groups and CCB

	BMI 18.5 to <24 (n=33259)	BMI 24 to <28 (n=37069)	BMI≥28 (n=16307)	Per 5-unit increase
**Complete RBBB**				
Case	206	251	111	
Incidence rate, No./10 000 person-y	5.80	6.37	6.41	
Model1	Ref	1.03 (0.86-1.24)	1.10 (0.87-1.38)	1.02 (0.90-1.16)
Model2	Ref	1.04 (0.86-1.25)	1.11 (0.87-1.40)	1.03 (0.90-1.17)
Model3	Ref	1.04 (0.86-1.25)	1.11 (0.80-1.42)	1.03 (0.90-1.17)
Model4	Ref	1.03 (0.86-1.25)	1.09 (0.86-1.39)	1.02 (0.90-1.16)
**Incomplete RBBB**				
Case	319	366	165	
Incidence rate, No./10 000 person-y	9.00	9.30	9.54	
Model1	Ref	0.98 (0.84-1.13)	1.04 (0.86-1.25)	1.06 (0.96-1.17)
Model2	Ref	1.03 (0.89-1.20)	1.16 (0.96-1.40)	1.13 (1.02-1.25)
Model3	Ref	1.03 (0.89-1.20)	1.15 (0.95-1.40)	1.13 (1.02-1.25)
Model4	Ref	1.03 (0.88-1.20)	1.15 (0.95-1.40)	1.13 (1.02-1.25)
**LAFB**				
Case	194	241	135	
Incidence rate, No./10 000 person-y	5.47	6.11	7.80	
Model1	Ref	1.04 (0.86-1.26)	1.39 (1.12-1.73)	1.24 (1.10-1.40)
Model2	Ref	0.99 (0.82-1.20)	1.26 (1.01-1.58)	1.17 (1.03-1.33)
Model3	Ref	0.99 (0.82-1.20)	1.25 (1.00-1.57)	1.17 (1.03-1.33)
Model4	Ref	1.00 (0.83-1.21)	1.29 (1.03-1.62)	1.19 (1.05-1.35)
**LPFB**				
Case	6	7	0	
Incidence rate, No./10 000 person-y	0.17	0.18	0.00	
**2nd1AVB**				
Case	5	3	1	
Incidence rate, No./10 000 person-y	0.14	0.08	0.06	
**Complete LBBB**				
Case	12	18	10	
Incidence rate, No./10 000 person-y	0.34	0.46	0.58	
Model1	Ref	1.31 (0.63-2.71)	1.72 (0.74-3.98)	1.33 (0.86-2.06)
Model2	Ref	1.37 (0.66-2.87)	1.91 (0.81-4.51)	1.42 (0.91-2.23)
Model3	Ref	1.37 (0.66-2.86)	1.90 (0.81-4.50)	1.41 (0.90-2.22)
Model4	Ref	1.30 (0.62-2.73)	1.72 (0.72-4.10)	1.34 (0.84-2.13)
**Incomplete LBBB**				
Case	8	13	6	

© 2023 Liu P et al. *JAMA Network Open.*

Incidence rate, No./10 000 person-y	0.22	0.33	0.35	
Model1	Ref	1.36 (0.56-3.28)	1.52 (0.53-4.38)	1.49 (0.88-2.51)
Model2	Ref	1.33 (0.55-3.25)	1.46 (0.49-4.30)	1.44 (0.84-2.46)
Model3	Ref	1.32 (0.54-3.23)	1.42 (0.48-4.19)	1.43 (0.83-2.44)
Model4	Ref	1.25 (0.51-3.07)	1.28 (0.43-3.82)	1.37 (0.79-2.38)

Abbreviations: Complete RBBB, complete right bundle branch block; Incomplete RBBB, incomplete right bundle branch block; Complete LBBB, complete left bundle branch block; Incomplete LBBB, incomplete left bundle branch block; LAFB, left anterior fascicular block; LPFB, left posterior fascicular block; 2nd1AVB, second-degree type 1 atrioventricular block.

Model 1 adjusted for age and sex.

Model 2 adjusted for age, sex, smoking, drinking, high salt intake, physical activity, high-sensitivity C reactive protein level, low-density lipoprotein cholesterol level, high-density lipoprotein cholesterol level, estimated glomerular filtration rate level, hypertension, and diabetes.

Model 3 adjusted for all the variables in model 2 and myocardial infarction during follow-up and heart failure during follow-up.

Model 4 adjusted for all the variables in model 3 and antidiabetic treatment, antihypertensive treatment and lipid-lowering drug.

© 2023 Liu P et al. *JAMA Network Open.*

eTable 3. Hazard ratio and 95% confidence interval for the association between different body mass index (BMI) groups and CCB using time-dependent cox proportional hazards model

	BMI 18.5 to <24 (n=33259)	BMI 24 to <28 (n=37069)	BMI≥28 (n=16307)	Per 5-unit increase
CCB				
Model1	Ref	1.14 (1.06-1.23)	1.34 (1.23-1.47)	1.14 (1.08-1.19)
Model2	Ref	1.13 (1.05-1.22)	1.31 (1.20-1.43)	1.13 (1.08-1.18)
Model3	Ref	1.14 (1.05-1.22)	1.33 (1.21-1.45)	1.14 (1.08-1.19)
Model4	Ref	1.13 (1.05-1.22)	1.33 (1.21-1.45)	1.14 (1.08-1.19)

Abbreviations: BMI, body mass index (calculated as weight in kilograms divided by height in 2 meters squared); CCB, cardiac conduction block.

Model 1 adjusted for age and sex.

Model 2 adjusted for age, sex, smoking, drinking, high salt intake, physical activity, high-sensitivity C reactive protein level, low-density lipoprotein cholesterol level, high-density lipoprotein cholesterol level, estimated glomerular filtration rate level, hypertension, and diabetes.
Model 3 adjusted for all the variables in model 2 and myocardial infarction during follow-up, heart failure during follow-up.

Model 4 adjusted for all the variables in model 3 and antidiabetic treatment, antihypertensive treatment, lipid-lowering drug.

© 2023 Liu P et al. *JAMA Network Open.*

eTable 4. Hazard ratio and 95% confidence interval for the association between different body mass index (BMI) groups and CCB, sensitivity analyses

	BMI 18.5 to <24	BMI 24 to <28	BMI≥28
CCB			
Excluded new-onset cardiac conduction block cases within the first 2 years of follow-up (n=861)			
Model1	Ref.	1.05 (0.93-1.19)	1.25 (1.07-1.47)
Model2	Ref.	1.04 (0.92-1.18)	1.23 (1.06-1.44)
Model3	Ref.	1.04 (0.92-1.17)	1.24 (1.06-1.44)
Excluded new-onset myocardial infarction during follow-up (n=1121)			
Model1	Ref.	1.07 (0.95-1.20)	1.30 (1.13-1.50)
Model2	Ref.	1.05 (0.92-1.19)	1.26 (1.08-1.48)
Model3[a]	Ref.	1.04 (0.92-1.17)	1.24 (1.07-1.44)
Excluded new-onset heart failure during follow-up (n=906)			
Model1	Ref.	1.05 (0.93-1.18)	1.27 (1.10-1.47)
Model2	Ref.	1.04 (0.92-1.18)	1.26 (1.08-1.46)
Model3[b]	Ref.	1.02 (0.91-1.16)	1.21 (1.04-1.41)

Abbreviations: BMI, body mass index (calculated as weight in kilograms divided by height in 2 meters squared); CCB, cardiac conduction block.

Model 1 adjusted for age and sex.

Model 2 adjusted for age, sex, smoking, drinking, high salt intake, physical activity, high-sensitivity C reactive protein level, low-density lipoprotein cholesterol level, high-density lipoprotein cholesterol level, estimated glomerular filtration rate level, hypertension, diabetes.

Model 3 adjusted for all the variables in model 2 and antidiabetic treatment, antihypertensive treatment, lipid-lowering drug.

Model 3[a] adjusted for all the variables in model 2 and heart failure during follow-up, antidiabetic treatment, antihypertensive treatment, lipid-lowering drug.

Model 3[b] adjusted for all the variables in model 2 and myocardial infarction during follow-up, antidiabetic treatment, antihypertensive treatment, lipid-lowering drug.

.

© 2023 Liu P et al. *JAMA Network Open.*

eTable 5. Hazard ratio and 95% confidence interval for the association between different body mass index (BMI) groups and CCB using Fine-Gray model

	BMI 18.5 to <24 (n=33259)	BMI 24 to <28 (n=37069)	BMI≥28 (n=16307)
CCB			
Model 1	Ref	1.06 (0.94-1.20)	1.26 (1.08-1.47)
Model 2	Ref	1.05 (0.92-1.19)	1.23 (1.05-1.43)
Model 3	Ref	1.05 (0.92-1.19)	1.22 (1.05-1.43)
Model 4	Ref	1.05 (0.92-1.19)	1.23 (1.05-1.43)

Abbreviations: BMI, body mass index (calculated as weight in kilograms divided by height in 2 meters squared); CCB, cardiac conduction block.

Model 1 adjusted for age and sex.

Model 2 adjusted for age, sex, smoking, drinking, high salt intake, physical activity, high-sensitivity C reactive protein level, low-density lipoprotein cholesterol level, high-density lipoprotein cholesterol level, estimated glomerular filtration rate level, hypertension, and diabetes.

Model 3 adjusted for all the variables in model 2 and myocardial infarction during follow-up, heart failure during follow-up.

Model 4 adjusted for all the variables in model 3 and antidiabetic treatment, antihypertensive treatment, lipid-lowering drug.

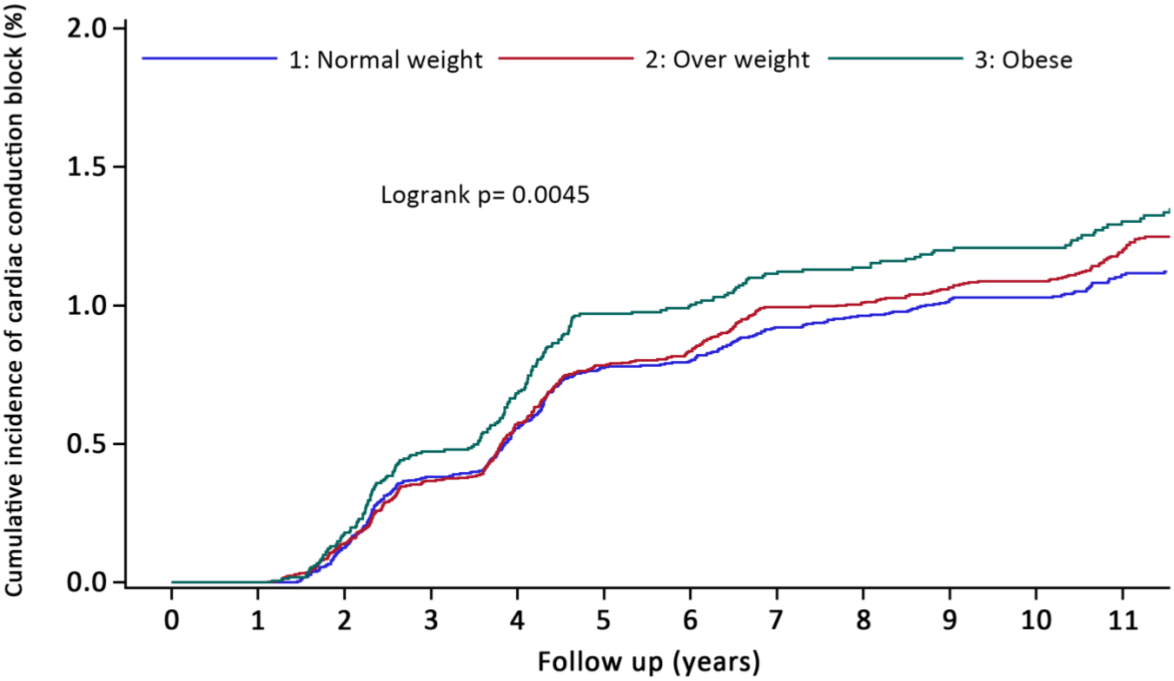

1	33259	33257	32913	32179	31525	30534	29849	28027	26951	24894	23816	19689
2	37069	37066	36668	35882	35087	33992	33275	31228	29972	27584	26337	21279
3	16307	16306	16119	15747	15439	14937	14623	13712	13120	12130	11575	9316

eFigure 1： Cumulative incidences for CCB. CCB is defined as the occurrence of any of the following: High-grade atrioventricular block, complete right bundle branch block, complete left bundle branch block, left anterior fascicular block, or left posterior fascicular block.

© 2023 Liu P et al. *JAMA Network Open.*

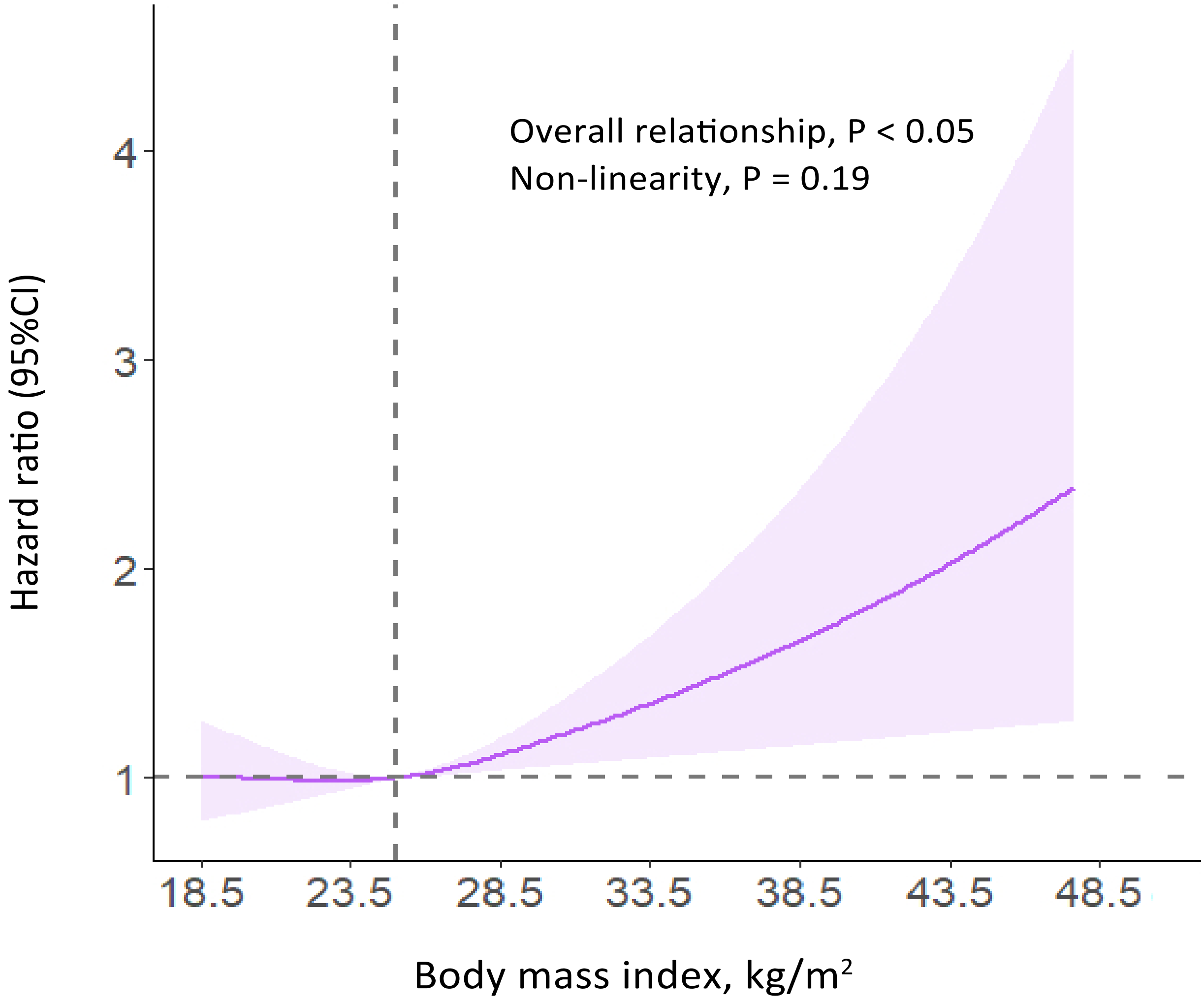

eFigure 2. Dose-response Relationship between BMI and CCB. Restricted cubic splines were constructed with three knots located at the 25th, 50th, and 75th percentiles of BMI. adjusted for age, sex, smoking, drinking, high salt intake, physical activity, high-sensitivity C reactive protein level, low-density lipoprotein cholesterol level, high-density lipoprotein cholesterol level, estimated glomerular filtration rate level, hypertension, diabetes, myocardial infarction during follow-up, heart failure during follow-up, antidiabetic treatment, antihypertensive treatment, and lipid-lowering drug.

© 2023 Liu P et al. *JAMA Network Open.*

eMethod 1. Definitions

Participants who developed myocardial infarction or heart failure during follow-up were identified and recorded this using the International Classification of Diseases codes (ICD-10) by trained personnel. Hypertension was defined as having a systolic blood pressure (SBP) ≥ 140 mmHg/diastolic blood pressure (DBP) ≥ 90 mmHg, or the current use of antihypertensive drugs. Diabetes was defined using a fasting blood glucose (FBG) ≥ 7.0 mmol/L or the use of antidiabetic drugs. Drinking was defined using a mean daily intake of 100 ml liquor (alcohol content ≥ 50%) for at least 1 year. Smoking was defined as smoking at least 1 cigarette per day during the preceding year. Physical exercise was defined as the taking of exercise ≥ 3 times per week for ≥ 30 min on each occasion. High salt intake was defined as the consumption of > 6 g salt/day.

© 2023 Liu P et al. *JAMA Network Open.*
