# Supplementary material for: Obesity and Cardiac Conduction Block Disease in China
Source: JAMA Netw Open. 2023 Nov 13;6(11):e2342831. doi: 10.1001/jamanetworkopen.2023.42831 (PMC10644217; doi:10.1001/jamanetworkopen.2023.42831)
Supplement: Supplement 2. — Data Sharing Statement [file jamanetwopen-e2342831-s002.pdf]

Data Sharing Statement

Liu. Obesity and Cardiac Conduction Block Disease in China. *JAMA Netw Open*. Published online November 13, 2023. doi:10.1001/jamanetworkopen.2023.42831

Data
Data available: No
